# Integrating Genetic Information into the Electronic Health Record: The Case of Adolescents’ Revelation of Misattributed Parentage

**DOI:** 10.1007/s11673-025-10448-1

**Published:** 2025-10-27

**Authors:** Sivan Tamir, Sivan Gazit, Tal Patalon

**Affiliations:** 1 Kahn Sagol Maccabi (KSM) Research & Innovation Center, Maccabi Healthcare Services, 68125 Tel Aviv, Israel; 2 Center for Health, Law and Ethics, University of Haifa, Haifa, Israel; 3 Maccabi Healthcare Services, 68125 Tel Aviv, Israel

**Keywords:** Genetic information, Electronic health record, Misattributed parentage, Genetic counselling, Health privacy, Autonomy

## Abstract

In the near future, the electronic health record (EHR) is likely to include patients’ genetic information, in addition to other clinical information and administrative data. This warrants some preparatory thought. Presently, in many jurisdictions, adolescents can self-access their EHR, as an acknowledgment of their relative autonomy in areas concerning their health. The foreseeable routine integration of genetic information into the EHR could result, inter alia, in adolescents’ inadvertent exposure to misattributed parentage findings emerging from genetic investigations, creating clinical challenges and ethical difficulties. The article reviews policy on the underexplored area of sharing genetic (non-)relatedness information with adolescents. It then considers the clinical perspective of adolescents revealing genetic non-relatedness through the EHR. Next, the article comparatively investigates adolescents’ health privacy and EHR-access laws and policies across several jurisdictions. After that, it analyses applicable ethical principles, such as minors’ right to know their genetic origins; parents’ (contentious) right to know that their child is genetically unrelated to them; the best interests of the child; and minors’ autonomy. Finally, we offer an implementable model in which the minor makes an informed choice of consent option, genetic results are segregated from other EHR data, differential access is applied to genetic information; and pre-disclosure medical counselling for adolescent patients is mandated.

## Introduction

The electronic health record (EHR), accessible to patients through patient portals, commonly includes clinical information (e.g., medical history, laboratory test results, diagnoses, medications), patient demographics, and administrative and billing data. Genetic information (GI) is not yet routinely included in the EHR globally but is expected to be introduced therein in the near future (Nestor et al. 2021; Jason 2021; Lau-Min et al. 2022), as the mainstreaming of genomics is increasingly expanding. GI that is likely to be available through the EHR— depending on local law and the policy of the health maintenance organization (HMO)—includes, for example, results from single gene tests for monogenic diseases, gene panel tests that examine multiple genes, and large-scale whole-genome or whole-exome sequencing. These broader tests analyse an individual’s entire genome or exome (protein-coding regions) to identify genetic variations known to cause specific inherited genetic conditions or increase the risk for such conditions.

Adolescents’ exposure to GI through their EHR is a particularly sensitive issue warranting attention. Typical circumstances in which minors are genetically tested include (a) diagnosing a hereditary condition, assessing the risk of developing such a condition, or identifying if the child is a carrier of a genetic mutation leading to such a condition; (b) polygenic risk score analysis to estimate a minor’s genetic susceptibility to a trait or disease; (c) prenatal testing for pregnant adolescents; and (d) direct-to-consumer (DTC) genetic testing, where adolescents or their parents seek GI independently.

In many jurisdictions, adolescents can self-access their EHR. Enabling pre-adulthood access to medical records is an acknowledgment of minors’ relative autonomy in areas concerning their health (e.g., to seek medical advice, get tested, and consent to certain medical procedures). It empowers adolescents and advances their transition to adulthood by allowing and encouraging them to participate in their healthcare management (Hagström et al. 2022b).

Apprehensions relating to adolescents’ independent access to their EHR generally focus on two key concerns: (1) adolescent confidentiality and health privacy (Hagström et al. 2022b) and (2) minors’ low medical literacy. That is, that by independently processing health information not communicated to them by their physician and without professional counselling, adolescents may poorly understand the information, misinterpret it, or detrimentally act upon it. Insufficient health literacy, emotional maturity or resilience could result in significant distress for adolescents (Lee and Holland-Hall 2021), potentially leading to physical and psychological harm. The foreseeable routine integration of GI into the EHR could exacerbate such concerns.

Imagine a minor undergoing genetic testing to diagnose a potentially hereditary condition, such as beta thalassemia intermedia, a recessive genetic condition commonly expressed during adolescence. Often, when a minor is found to be positive (i.e., carrying the genetic mutation), parents and siblings are tested as well, both for their own health and for future reproductive needs. If the results of such genetic investigation documented in the testees’ EHR, indicate that the parents are non-carriers, a finding of misattributed parentage (MP) (interchangeably used with “non-relatedness”) incidentally emerges.

MP circumstances include misattributed paternity (where the presumed father is not the genetic father as a result of extradyadic relations or sperm mix-up in intrauterine insemination (IUI) or in-vitro fertilization (IVF)); non-maternity (a less common situation due to the intended mother’s egg being mixed up in IVF); or situations where neither parent is genetically related to the child (IVF embryo mix-up). Reported rates of misattributed paternity vary erratically, ranging between one to ten per cent (Lawton et al. 2023), or 0.8 to thirty per cent (Bellis 2005; Hercher and Jamal 2016; Lowe et al. 2017), with scarce data on the rarer incidence of non-maternity/MP (Botkin et al. 2015).

And so, by accessing GI independently available through the EHR, the adolescent patient could now, in addition to the risk of learning predictive GI about her health, incidentally reveal MP (Grebe et al. 2020), with consequent psychosocial implications.

Studies show that most parents prefer not to disclose genetic non-relatedness to their children, for reasons associated with the welfare of the child, privacy of parents, and maintaining familial harmony (Ravitsky 2017). MP (non-)discovery generates myriad ethical and clinical issues for adolescents. Due to space constraints, we shall focus on those directly tied to the incidental finding of MP, setting aside related issues such as the appropriate timing of genetic testing in minors and the use of predictive genetic testing in this population.

The paper is constructed as follows. We initially review the policy on sharing genetic relatedness information with adolescents. We then consider the clinical perspective of adolescents learning of genetic non-relatedness through the EHR. Next, we comparatively investigate adolescents’ health privacy and EHR-access laws and policies across several jurisdictions. After that, we analyse applicable ethical principles, such as minors’ right to know their genetic origins; parents’ (contentious) right to know that their child is genetically unrelated to them; the best interests of the child; and minors’ autonomy in the context before us. This paper provides a theoretical, descriptive account of the potential implications of integrating GI into the EHR, particularly as it relates to the risk of adolescents inadvertently revealing MP. Given the foreseeable benefits of such integration, primarily the promise it holds for advancing personalized medicine (Tamir et al. 2024), our position is one that supports such a practice but warrants caution at the same time. Recognizing the inevitability of such development, we therefore conclude by suggesting an implementable model with differential access to GI, which inter alia involves the minor informedly selecting a consent option and mandates pre-disclosure medical counselling for adolescent patients.

## Policy on Sharing Genetic Relatedness Information

This is a relatively underexplored area. Most studies investigating sharing medical information with patients and their relatives focus on the disclosure of health-related information (genetic testing results, distressing diagnoses). Also, when non-relatedness is being explored, focus is typically placed on disclosing a non-paternity finding to the father, with little attention given in the literature to disclosure of MP/non-paternity to the child.

As no clear directives on policy exist, a policy of de facto non-disclosure is being practiced (Hercher and Jamal 2016, 36). The few professional guidelines in place are contradictory, typically addressing disclosure of non-paternity to spouses rather than to children (Hercher and Jamal 2016). Notably, policies and guidelines mentioned in this section only apply to healthcare providers and do not impose legal or moral obligations on parents.

In 2013, the American College of Medical Genetics and Genomics (ACMG) published its “Points to consider for informed consent for genome/exome sequencing” (ACMG 2013), briefly addressing non-health related ancestry information, while failing to specifically address MP findings. No specific attention was given to minors in this context.

In 2015, the American Society of Human Genetics (ASHG) published its somewhat paternalistic position on the ethical, legal, and psychosocial implications of genetic testing in children and adolescents, recommending that “health-care providers avoid disclosure of misattributed parentage unless there is a clear medical benefit that outweighs the potential harms” (Botkin et al. 2015, 15). Arguably, restricting MP disclosure to settings with clinical benefit or actionability ignores the immense value of knowing one’s (or one’s child’s) authentic genealogy. In other words, such paternalism could be questionable, given the increasing weight ascribed to one’s right to know one’s genetic origins (addressed below) and to parents' right to know their children’s genetic relatedness to them.

The weaknesses of a paternalistic non-disclosure policy should also be considered against the backdrop of children’s and young persons’ technological savviness (digital nativity). The potential for MP to be proactively revealed by adolescents increases with the availability of DTC genetic ancestry testing, easily accessible not only for parents suspecting non-relatedness but for “inquisitive” adolescents as well.

Others will claim that disclosing incidental information that may have no clinical relevance to the minor could be more harmful than beneficial and it is not the physician’s responsibility to decide on such disclosure, thereby justifying a protective approach.

Another point is that disclosure of one’s procreative circumstances is typically reserved to parents (Ravitsky 2017) for a host of rationales. Revealing MP information by mere access to one’s EHR will not only circumvent the parental prerogative in this area but may also compromise the minor’s psychosocial well-being as analysed below.

Another relevant policy document is ACMG’s (Deignan et al. 2020) “Points to consider when assessing relationships (or suspecting misattributed relationships) during family-based clinical genomic testing.” It focuses on MP, disclosed following parents-child (proband) “trio” testing for identifying DNA variations known to cause specific inherited genetic conditions. These guidelines state that non-relatedness information should not be considered a reportable incidental/secondary finding in the same way as pathogenic variants might be. They consequently narrowly determine that from a clinical framework, there is *no duty* to disclose non-relatedness of genetic samples, overlooking the complexities of the issue and the misaligned interests of the different trio testees in the context of MP (non-) disclosure. This is particularly striking in view of the stated fact that “[a]n accurate trio-based analysis cannot be completed using samples that are not biologically related” (Deignan et al. 2020, 1285). How then, will the clinical geneticist explain to the family the non-completion of the test, without revealing genetic non-relatedness between child and parent/s? Clearly, the policy’s recommended wording to be used in the informed consent form, that “the relatedness assessment determines that one or more of the samples is *not informative*” (emphasis added by authors) (Deignan et al. 2020, 1286), is rather vague, particularly for lay patients. In any case, when leaning towards MP disclosure, laboratories and clinicians should operate under the assumption that all members of the parents-child trio being tested may have access to the minor’s report.

Lastly, the ACMG statement (Grebe et al. 2020) on “[t]he interface of genomic information with the electronic health record” recommends that genetic testing results accessible through patient portals be understandable and utilizable for the reasonable patient and that the “need for clinical interpretation and counselling related to the results and potential for patient harm” is taken into account in providing access to such information (Grebe et al. 2020, 1434). We fully support this position and recommendation, and they are reflected in the practical model suggested below.

## The Clinical Perspective

Studies on adolescents’ access to their EHR via patient portals cite various benefits, such as enhanced patient-provider communication, ease of transition into adult care (particularly significant for adolescents with chronic health conditions), reduced anxiety, improved involvement in their own health management, and empowerment (Lee and Holland-Hall 2021; Nielsen et al. 2023; Hagström et al. 2024). Various concerns have also been raised. Apprehensions include misinterpreting health information in the EHR, relying on it inappropriately and taking harmful action as a result; threats to adolescents’ medical confidentiality and privacy, which could discourage them from seeking needed care; and the accidental disclosure of sensitive parental health information—such as conception circumstances (e.g., via IVF) or information indicating adoption status previously unknown to the minor (Lee and Holland-Hall 2021; Nielsen et al. 2023).

From a clinical perspective, a key issue is the physician-adolescent patient relationship. In the setting before us, where adolescents have independent (potentially unrestricted) access to their EHR, which includes GI, they are likely to discover genetic conditions (e.g., being carriers of a genetic mutation causing disease), as well as GI indicating MP, by themselves. Facing such a revelation without professional mediation, may cause the minor significant anxiety and emotional burden. It could also have significant trust-shattering effect both for the minor-parent relationship, and for the physician–patient one.

Learning that one’s physician had known about this life-altering fact yet chose not to disclose it to them, while perhaps sharing this fact with their mother/parents, might feel conspiratory and dishonest, violating the fundamental transparency and honesty principles.

Good medical practice cannot be delivered without trust, as trust both encourages and relies on disclosure to patients, whereas *mis*trust affects patients’ compliance with medical advice and consequent adherence to treatment. Physician’s transparency and honesty are also a trust-building block, with particular significance within power asymmetries between physician and patient. And so, where a minor discovers their physician has been non-revealing, not only will that specific relationship be in danger—leading to patient’s non-cooperation with medical management, with potentially detrimental consequences to their health—but it may harm the individual’s entire trust in the medical system.

Another related medico-legal issue is that of medical privacy, significantly affecting clinical practice. We shall discuss this in depth in the section below.

Having identified the main clinical pillars of trust, transparency and confidentiality, and allowing for appropriate care and support systems for the (minor) patient, we ought to consider their increased complexity in the context of MP.

For instance, adolescents’ access to their EHR containing incidental MP information may pose clinical challenges for the relationships between the treating physician and the minor patient’s relatives, who may also be the physician’s patients. Consider a situation in which a minor discovers a predisposition to a malignant disease as well as MP (e.g., non-paternity) and subsequently requests the physician *not* to reveal such information to their (apparently) non-biological father. That minor is now subjected to screening and other tests as part of preventive medical practice. How should a physician react when the father inquires why he or other siblings are not subjected to these tests? While the physician is obliged to maintain the minor patient’s confidentiality, they may also have a duty of care towards the patient’s family members (particularly if they too, are the physician’s patients). As genetically non-related family members do not share the predisposition to genetic disease, the physician should not unjustifiably subject them to unnecessary, occasionally invasive tests, carrying physical and emotional burden. Additionally, the physician is ethically obliged not to misuse limited medical resources.

Thus, knowing one’s genetic-relatedness—that is, identifying *who is* and (no less important) *who is not* one’s blood relative—could be pertinent for taking preventive strategies, for diagnostic genetic investigations, and for reproductive decision-making (Ravitsky 2017).

Wright et al. (2019, 4) argue that the decision whether to disclose MP in a clinical setting should be determined according to its clinical significance. Consequently, non-disclosure in unlikely to be justified in cases “where inheritance patterns are crucial to the interpretation of a result, or for the delivery of clear and honest information about reproductive choice.”

While it’s understandable that clinicians need to draw boundaries—and have limited tools for managing patients’ incidental discovery of MP—this clinical perspective may be too narrow, overlooking other important patient interests that justify disclosure, such as the right to know one’s genetic origins (addressed below).

Finally, we address two practical clinical difficulties. First, one must acknowledge that most physicians have not been trained to provide counselling for patients who have learnt of MP. Whereas delivering bad or difficult news in medicine is taught from early training stages, counselling patients on dealing with MP requires a multi-dimensional (psychological, social and culture sensitive) approach—a task for which physicians are not particularly educated. Allowing independent access to such information through EHR will therefore necessitate appropriate training programs for primary care physicians, at least.

Another practical concern is that paediatricians often view adolescent access to patient portals with some scepticism. They cite barriers such as time-consuming decision-making, limited technological and staffing resources, and inconsistent implementation—largely due to “absent guidelines and vague laws” (Hagström et al. 2022b, 12).

We now move to examine the ethico-legal issue of adolescents’ health privacy and access to their EHR.

## Adolescents’ Health Privacy and Access to the EHR—A Comparative Perspective

Three closely related yet distinct key issues involve minors’ legal capacity with regard to healthcare. These are their 1) capacity to consent, 2) control over the disclosure of health information to third parties, and 3) self-access to their EHR. We are chiefly focused on the latter two.

As a rule, patients have a right to access their medical information. Unless specifically excluded, this includes *genetic* information (especially such that is health-related). For instance, under the U.S. HIPAA Health Privacy Rule, the only material exception to individuals’ *right of access* to protected health information within the EHR is access to psychotherapy notes,^1^ which are kept separately from the medical record, inaccessible to *both* parents and children (Hales 2020).

Interestingly, ACMG’s “Points to consider …” notes that [parent-proband] trio testing “strains traditional concepts of medical record privacy,” as GI about both parents could be included in the child’s health record (Deignan et al. 2020, 1287). This could not only lead to adolescents learning of genetic (non-)relatedness but equally affect parents’ privacy by potentially exposing them to such information about one another through access to their child’s EHR.

Policies on when minors/adolescents can *self-access* their EHR vary widely across jurisdictions, typically ranging between twelve and eighteen years or older (Nielsen et al. 2023) (see Table 1). As children turn into adolescents, issues around their health privacy start to crop up. Minors’ (relative) right to medical privacy/confidentiality is an exception to the general rule that parents—legally recognized as their children’s personal representatives—have the right to know, and consequently—to have access to their minor child’s protected health information (Hales 2020).

**Table 1:** Adolescents’ access to the EHR—A comparative look

Country	Age of adolescent’s self-access to EHR	Age of (fully/partially) blocking parental access to EHR	Exception to right of access—or—differential access to health information	Access to genetic information in the EHR	Age of minor’s consent to medical care (without need for parental/guardian consent)
U.S	12–14 12—with parental consent; 13—without parental consent (Carlson and Goldstein 2020)	Restrictions on parental access to adolescent’s EHR—varying between none, limited (teen proxy) access/differential access (e.g., sensitive information redacted), and complete withholding of EHR access	Psychotherapy notes	N/A	18 Exception: minors sufficiently mature to make their own healthcare decisions (“mature minor doctrine”^a^)
U.K	11 or 12	13—minors are considered to have the capacity to give or refuse consent to parents’ access to their EHR 11–16—parents may be allowed *proxy access* 16—parental access blocked	Information that would *cause minors serious harm;* and information about others without their consent	N/A	16, or younger – depending on the minor’s capability to appreciate the nature and implications of the treatment/diagnosis, i.e. “Gillick competent” (BMA 2024)
Sweden	16 Adolescents can apply for earlier self-access Electronic ID required (available at 8 years old)	13 13–16—gap period: no access to either parent or child Parents can apply for access outside of the default	No sensitive health information exempted from parental access Exception: one region exempting psychiatry notes from availability in the national EHR service	N/A	No fixed minimum age requirement. A case-by-case policy, depending on the maturity of the child (EU Agency for Fundamental Rights 2017)
Norway	16 Electronic ID required (available at 16 years old)	12 Exception: where minors’ health information is required for fulfilling parental duties, parents may get the information between age 16 and 18 (with notification to the adolescent) (Helsenorge 2023)	No sensitive health information exempted from parental access	N/A	16 Exception: refusal to treatment—18 (Helsenorge 2023)
Finland	No lower age limit for EHR self-access Access enabled upon acquiring an electronic ID	Parental access possible by default until age 18 (but could potentially be lost at the age of 10)	No sensitive health information exempted from parental access	N/A	18 (FRA 2017)
Estonia	No lower age limit for EHR self-access Access enabled upon acquiring an electronic ID	18	No sensitive health information exempted from parental access	N/A	No fixed minimum age requirement. A case-by-case policy, depending on the maturity of the child (FRA 2017)
Israel	N/A (18)	N/A (18)	–––	N/A	18 Exceptions: 14—routine medical care; abortion – any age; HIV testing (at age 14—with doctor’s permission, or younger—permission of doctor & social worker required);^b^ psychiatric hospitalization—15; advanced directives—17; 16—genetic testing (with legal representative’s consent)
Australia	1—My Health Record [MHR]—adolescents’ sole control and access	14 Authorized representative(s) removed automatically from the adolescent’s EHR (ADHA “My Health Record for teens”)	Adolescents have discretion on: 1) Whether to give (whole/selective) access to their MHR, to *parents or other nominated representatives* 2) Restricting *healthcare provider access* to their record, or to specific documents therein	N/A	14—simple healthcare treatment; 16—adult-like competence to consent to medical treatment (raisingchildren.net.au. 2023)

^a^Minor patients possessing the maturity and capacity to understand the nature and purpose of the proposed treatment, are capable of providing consent

^b^Law for the detection of AIDS viruses in minors, 5766–1996 [in Hebrew]

Adolescents’ health privacy laws and policies—similarly varying between jurisdictions (Hagström et al. 2022c)—allow, inter alia*,* the blocking of parental access or restricting it under various models in different adolescence stages: For example, United Kingdom—at age sixteen (NHS (National Health Service) 2024a, 2024b) or thirteen where the minor has capacity to refuse consent to parents’ access to their EHR (House of Commons Library 2022), Australia—at fourteen (Bogle 2018); Sweden and Norway—at sixteen (Hagström et al. 2022c) (see Table 1).

In the United States, adolescents’ access to their EHR through patient portals is typically enabled between the ages of twelve and fourteen (Lee and Holland-Hall 2021). Parental access to adolescents’ EHR vary between no restrictions, partial/differential access (with sensitive information redacted), and complete withholding of access (Ancker et al. 2018) (see Table 1).

A comparative study of minors’ (and parental) access to the EHR, conducted across Sweden, Norway, Finland, and Estonia (considered forerunners in patient accessible EHR implementation) found that minors’ self-access was the same (age sixteen) in Sweden and Norway, while in Finland and Estonia, with no lower age for minors obtaining access to their EHR, self-access depends on acquiring an electronic ID (Sweden—age eight; Norway—age sixteen) (see Table 1). Interestingly, the study found that “[n]one of the studied countries exempted any potentially sensitive health information by default from parent access” except for one Swedish region which exempted child and adolescent psychiatry notes from the national EHR service (Hagström et al. 2022c, 497).

Also, in Sweden, where parental access to their child’s EHR is blocked by default at thirteen, there is a gap period until adolescents’ self-access at sixteen years, during which *no one* has access to the child’s EHR (unless the adolescent opts for providing her parent(s) with continued access to their EHR through an administrative process) (Hagström et al. 2022a).

In Israel, minors do not have self-access to the EHR, and parents are only barred from access once their child turns eighteen. Paradoxically, while Israeli adolescents are required to give consent to be genetically tested or to participate in genetic research from age sixteen and upwards,^2^ and terminally-ill adolescents can provide advance directives at the age of seventeen^3^—they are not entrusted with access to their EHR before adulthood (age eighteen).

In Australia, the *Privacy Act 1988* (Commonwealth), pertaining to personal and health information held by private sector providers, applies throughout its states (Bartholomeusz 2021). Public sector agencies, and some private sector providers, are covered by states’ and territories’ local laws on privacy and health records. Australia’s national digital health record, My Health Record, allows adolescents’ sole control and access at age fourteen (Australian Digital Health Agency (ADHA) “My Health Record for teens” no date; Beaton et al. 2021), along with discretion on whether to give access (wholly or selectively) to parents or other nominated representatives (ADHA “Nominated representatives” no date). This does not happen automatically but requires the minor to proactively turn on access settings (Bogle 2018). Adolescents can also restrict healthcare provider access to their record, or to specific documents (with the caveat that this “may limit a healthcare provider’s ability to access your medical information”) (ADHA “Restrict access” no date; see Table 1).

In the United Kingdom, according to the General Medical Council (GMC) guidelines, young people with capacity not only have the legal right to access their own health records, but can also allow or prevent access by others, including their parents. In Scotland, adolescents as young as twelve are legally presumed to have such capacity. However, the guidelines stipulate that minors “should not be given access to information that would cause them serious harm or any information about another person without the other person’s consent” (GMC 2023a, s. 53). This stipulation constricts children’s access to GI indicating MP in two ways. First, significant harm could be caused where such information prompts an “identity shock” (Lawton et al. 2023), undermining the child’s narrative identity, and disrupting intra-familial relations. Second, biological parentage information—by definition—involves information about another person. Therefore, disclosure of such information may effectively be barred—absent the consent of the relevant parent/s.

To conclude this part, there are two points to be made: First, after comparatively reviewing adolescents’ health privacy and access to the EHR across several jurisdictions, we found no specific regulatory reference to date, to minors’ access to their GI via the EHR/patient portals. This is no doubt due to the novelty of the topic. Second, the consideration of minors’—and particularly adolescents’—health privacy is not merely contingent on formal access to the EHR. The constant emergence of new health information technologies (e.g., telemedicine and health apps), as well as DTC genetic testing, accessible to both parents and adolescents, generates EHR-independent health information, with such platforms facilitating healthcare access and communication and creating new challenges for protecting minors’ privacy (Agostino and Toulany 2023).

We shall now analyse additional ethical issues arising from the incidental finding of MP, as a consequence of integrating GI into adolescents’ EHR.

## Ethical Analysis

As we stated at the outset, recognizing the inevitability of GI being integrated into the EHR and the risks of revealing genetic non-relatedness within the family that come with such a step, we deem such a practice to warrant caution. In this section we identify and analyse ethical considerations relevant to the changing architecture of the EHR, specifically for jurisdictions allowing minors’ access to it.

### The Minor’s Right to Know Her Genetic Origins

Arguments supporting disclosure of (non-)paternity findings generally “center on issues of a patient’s right to know, avoiding paternalism, and the duty of physicians to be truthful” (Botkin et al. 2015, 15).

The right to know one’s genetic origins, as clearly described by Ravitsky, relates to at least three aspects: the medical/clinical aspect; the personal identity aspect; and the relational aspect, i.e., the right to know one’s authentic genetic progenitors (Ravitsky 2017).

Arguably, knowing one’s genetic origins is vital for individuals’ physical and psychosocial well-being and instrumental to their autonomy (Ravitsky 2017). We have briefly addressed above the physical (clinical) perspective in the context of MP. As for the psychosocial perspective, while minors *inadvertently discovering* MP by accessing GI in their EHR could suffer psychological harm and face disruption of familial relationships–one’s true genetic origins are an essential component of their narrative identity, self-understanding, and self-determination. Consequently, concealing them from people, could carry personal and familial repercussions.

Support for the significance attributed to knowing one’s genetic origins can be found, for example, in the U.K. policy on gamete-donor anonymity. After having abolished it in 2005, the Human Fertilisation and Embryology Authority (HFEA) now takes concrete measures (i.e., updating donors’ contact details) to allow donor-conceived individuals to realize their right to know, and to attempt contact with, their “genetic progenitors” (Ochuko 2023). It has even recently launched a public campaign (#WhoIsMyDonor) encouraging donors to update their information, as gamete donor anonymity is to be lifted in the United Kingdom for the first time (Van Kerckvoorde 2023).

### The Parent’s Right to Know(?)

When MP is incidentally revealed through minors’ genetic testing, various questions emerge: Do parents have a right to know? If so, whose responsibility/duty is it to share non-relatedness information with them (Rhodes 1998)? Is it the physician’s or the genetic counsellor’s duty, or is it the minor-proband’s duty? And does non-relatedness information reside under the minor’s right to privacy and medical confidentiality?

It is not self-evident in the setting before us that parents have a recognized right to know about the newly discovered genetic non-relatedness to their child, particularly in the case of adolescents. It is also impractical to treat the “parents” as a single unit with identical interests, for it may be the case that the mother is in fact aware of non-paternity, while the father is ignorant of it (making the right to know relevant to the father alone).

This setting potentially invokes a triangular conflict between the physician/genetic counsellor, the minor patient, and her relatives. Most relevant here, are the following conflicts:

- between the child’s right to health privacy and the parent/s’ right to know the genetic origins of their progeny, broadly construed as part of their reproductive autonomy; and
- between the minor’s health privacy and the parent/s’ (and siblings’) right to health, emerging where, e.g., non-relatedness information could spare family members’ unnecessary testing and surveillance. In this not-strictly-health-related setting, the parent/s’ interest in knowing may not easily trump the minor’s right to health privacy.

Notably, in jurisdictions where parental access to their children’s EHR is blocked at some stage (see Table 1), sharing sensitive genetic-(non)-relatedness information with (blocked) parents will probably require the adolescent’s consent (Hagström et al. 2022b). Such stipulation could be medically and ethically problematic, as it will keep (one, or both) parents ignorant of the genetic non-relatedness. They may consequently, for example, continue to pursue a futile genetic investigation for themselves or for their other children (not genetically related to the minor proband), with all the anxieties and burden it entails.

The ethico-legal conundrum of sharing GI with relatives, is a complex issue in its own right, and is beyond the scope of this paper. However, we offer a few points to consider in the context before us:

1. The literature and case law on this issue are typically associated with health-related information (e.g., indicating a hereditary genetic condition),^4^ whereas in the case of MP we are dealing with genealogical information, albeit such that may have medical consequences for (non-)relatives.
2. The literature and case law on this issue typically focus on obligations to share genetic information with biological relatives, whereas here we consider biological *non*-relatedness. The severance of *presumed* biological ties may render the moral justification for sharing the genetic information redundant.
3. Drawing on lessons from the more common case of sharing *health-related* GI with *biological relatives* (e.g., *ABC v St George’s* 2015, 2020), we suggest that the genetic counsellor’s duty of care towards third parties (the parents and siblings) could be satisfied by informing the minor patient that such information could carry medical implications for her non-biological parents or non/partially-related siblings. The main clinical implication would be *avoiding* unnecessary genetic investigations, screening, and other preventive steps, which could result from the minor-proband’s genetic investigation.
4. As for the minor, legally, her consent for sharing non-relatedness information with her family will be required, since presumably, the overriding justification for disclosing such information to relatives to prevent *a serious risk* of harm (Lucassen and Gilbar 2018), does not apply here. Nonetheless, it could be argued that the minor has a *moral* duty, within the *ethics of care*, to share such information with her relatives to prevent harm (Gilbar 2016), unless she deems the risk of psychologically unsettling her parent/s or fracturing the family to overweigh the benefits of knowing about the MP finding.

Lastly, it remains an open question whether procreative circumstances should strengthen or weaken parents’ right to know in this context. Namely, whether in more “neutral” reproductive contexts (e.g., IVF) it could be ethically more complex to intentionally keep parents ignorant of the MP findings, potentially indicating embryo/gamete mishap, than in natural procreation cases, where non-relatedness could indicate infidelity. Although, it could be the case that neither genetic counsellor, nor the minor herself, are privy to the said circumstances.

### The Right Not to Know

Mirroring the right to know one’s genetic origins is the genetics-specific right *not* to know, referring to individuals’ right to opt for “genetic ignorance” (Takala 1999) when faced with the option of learning GI about themselves or their blood-relatives. While (in)famous for supporting ignorance in medical genetics contexts, such a right can be extended to genetic genealogy information. Namely, recognizing that minors have the right, as part of their autonomy and self-determination, not to know such information about themselves.^5^

Evidently, respecting the adolescent’s purportedly autonomous choice of not knowing GI, particularly such that may be extremely distressing (e.g., MP), places the physician or the genetic counsellor in conflict with their patient, specifically as it relates to their legal duty of care, and their ethical duties of beneficence and non-maleficence and the duty to respect their minor patient’s (contextually limited) autonomy. In medical contexts, the harms of receiving certain medical information, particularly when there is no clinical actionability, are weighed against the foreseeable risks/burdens of maintaining medical ignorance (Armitage 2023). In genealogy-related contexts, the harms of (not) knowing are much harder to evaluate.

This is of particular significance in view of the fatal “inability to ‘undo’ the knowledge” learnt from genetic testing (European Society of Human Genetics 2009), relating not only to health-related information, but also–if not more strongly–to genealogy-related information. Such consequence should be considered by healthcare professionals and explicitly divulged to both parents and minors (commensurate with the minor’s ability to understand), *prior* to testing the latter; particularly, where GI is accessible to adolescents through their EHR.

### The Best Interests of the Child

Notwithstanding supportive arguments for disclosure, the right *not* to know should routinely be considered, in conformity with the best interests of the child principle—the principle that the child’s prime interests should be “taken into account as a primary consideration in all actions or decisions that concern or affect children” (European Commission no date). The best interests of the child principle is a primary (context-dependent) objective of society and of the family unit in particular (Hercher and Jamal 2016).

Two key categories of child interests exist in the context before us: a) health-related interests; and b) psychosocial interests.

The (U.K.) GMC guidelines stipulate that “[a]n assessment of best interests will include what is *clinically* indicated in a particular case [emphasis added by authors],” taking into consideration, inter alia, the views of the minor and her parents, their cultural, religious or other beliefs and values, and an assessment of which choice will least restrict the child or young person’s future options (GMC 2023b, s. 12). So, although the guidelines address clinical aspects of healthcare, one could assume that this would equally, if not more forcefully, apply to genetic genealogy information accessible to the minor via the EHR.

From the health perspective, knowing one’s (authentic) family medical history is significant for diagnosis, health-risk assessment, and reproductive decision-making (Hercher and Jamal 2016). From the psychosocial aspect, timely disclosure could be less destructive than shielding the minor from knowledge about her non-relatedness to her parent/s, and deferring disclosure until she reaches adulthood, or is closer to the age of majority. Those opposing disclosure cite potential violence or abandonment*,* which are contrary to the best interests of the child and the family as a whole (Wright et al. 2019).

Hercher and Jamal elegantly criticize the often-made argument that nondisclosure in the case of misattributed paternity is essential for preserving the family unit and child-parent relationship. According to them, the argument mistakenly relies on the assumptions that a) disclosure would be detrimental to the family, while non-disclosure would protect it, and b) the particular family’s parent–child relationship is the child’s “best chance at a happy home life” (Hercher and Jamal 2016).

But with empirical research on the psychosocial effects of MP disclosure being scant (Lowe et al. 2017; Wright et al. 2019), caution is warranted in determining whether knowledge about MP is indeed consistent with a minor’s best interests.

So, although undesirable for coherent policymaking, as well as impracticable for the seamless integration of GI into the EHR, we need to consider both the merits and the failings of such disclosure, after identifying the best interests of the (particular) minor in being exposed to GI indicating MP. This could require the segregation of health-related and genealogy GI within the EHR, as suggested below.

### A Child’s Right to an Open Future

Another typically raised concern in this context, is that of the *child’s right to an open future,* as dubbed by Feinberg (1980). This is a collective term for children’s adult-like autonomy rights that cannot be exercised by the child until her decision-making capacity and maturity are more fully formed, and are thus kept in trust by parents/guardians. However, this right is often criticized. Garrett et al. (2019), for example, suggest that the relational value, or the direct relationship between learning of MP and the purported child’s right to an open future, is not axiomatic. They also challenge the quantitative reading of this right as a maximal ideal that seems impossible to satisfy. We agree with this critique. Given the incalculable and elusive nature of the concept of “open future” and it being child/family-specific, it is impracticable to predict whether a minor’s revelation of MP would either open or constrict her future.

### Minor Autonomy

It is a mainstream position in the modern liberal era that minors’ views should be respected, and their voice and choices taken into account on their pathway to autonomous decision-making.

Making GI available to adolescents through patient portals is a manifestation of respect for their autonomy by promoting self-governance in exclusive contexts pertaining to minors’ healthcare and bio-genetic ties.

Minorautonomy (a portmanteau of “minority” and “autonomy”) is an exception to parents’ “primary ethical and legal responsibility for their children’s healthcare” (Ancker et al. 2018, 1593), reflecting minors’ dynamic transitory autonomy, with autonomy-constraints being gradually lifted as adolescents become *quasi-competent* agents (Tamir 2016). It is a key tenet in the child’s self-shaping and self-determination processes and therefore, to cite the American Medical Association (AMA) Code of Medical ethics on Confidential Care for Minors (2012): “Physicians who treat minors have an ethical duty to promote the developing autonomy of minor patients by involving children in making decisions about their health care to a degree commensurate with the child’s abilities.”

While it is generally assumed that children and pre-adolescents cannot adequately understand clinical information, some researchers maintain that “children are capable of full informed consent from the age of 12” (Hagström et al. 2022c, 498). Arguably, handling newly (incidentally) acquired knowledge about one’s MP requires substantial emotional maturity and well-adjustedness—something that not even experienced adults necessarily possess.

Notwithstanding their psycho-emotional preparedness for coping with news about non-paternity/maternity, the autonomy of adolescents verging on adulthood should also be evaluated against the realistic backdrop of adolescents nowadays being able to independently research their genetic origins relatively easily through online genetic relative-finder services.

Minorautonomy and minors’ digital nativity, however, could be curbed by their low medical literacy (mentioned above), making it challenging for them to understand and process health information and navigate their way through complex health systems and the EHR (Beaton et al. 2021). As Beaton et al. (2021) note, [adolescents’] increased access to health information and digital health resources navigation, do not necessarily result in increased health literacy. However, studies that investigated adolescents’ views on accessing their EHRs found a strong interest in access among this group (in contrast to their reportedly relatively low actual use of patient portals). They also found that accessing their EHR is considered useful by adolescents and, particularly for (but not limited to) ill minors, enhances their knowledge about their health, has them better engaged with their healthcare (inter alia, by allowing them to ask informed questions), empowers them, and reduces anxiety (Hagström et al. 2022b; Hagström et al. 2024).

After concluding our clinical and ethical analyses, we use insights therefrom to delineate a desirable model for appropriately integrating GI into the EHR, taking into account MP-related complexities discussed above.

### A Suggested Model

For jurisdictions where minor patients have a right to access their medical information through the EHR (see Table 1), we propose the following model.

#### Nuanced Consent Options

As we have suggested elsewhere (Tamir et al. 2024) vis-à-vis adults revealing MP through the inclusion of GI in the EHR, at the point of registration for a patient portal—and again before any genetic testing is conducted—patients should be required to select one of the following mutually exclusive consent options:

- accessibility to all health-related GI and (medically derived) genetic genealogy information, including raw genetic data and (presently) non-interpretable GI pertaining to the patient.
- accessibility to strictly *clinically actionable* health-related GI pertaining to the patient.
- accessibility to medically derived, incidentally inferred genetic *genealogy information*.
- accessibility to strictly *clinically actionable* health-related GI pertaining to the patient, as well as to medically derived, incidentally inferred genetic *genealogy information*; and
- non-exposure to genetic information (remaining genetically ignorant).

These options will equally apply to adolescents, *mutatis mutandis,* and will be clarified in an explanation sheet, to ensure their understanding of the ramifications of the selected consent choice, to support informed consent.

#### A Context-Dependent Case-by-Case Approach

Given the rich array of non-relatedness revelation circumstances, we believe that in the event that genetic testing results indicate MP, a context-dependent approach, similar to what has been advocated in other research (see e.g., Lowe et al. 2017), should be applied to the case of minors. The context consideration will take into account parental interest in (not) knowing, the adolescent’s interest in knowing, and family-specific effects of MP disclosure. Such a context-sensitive approach may invite, for example, a distinction between disclosure of “neutral” cases of MP, say, following gamete or embryo mix-up during IVF treatment (where the fault is external to a couple’s relationships) and MP disclosure in natural procreation cases, such as non-paternity, which are emotionally charged and potentially more disruptive to relationships.

Support for a case-by-case approach can be found in the AMA guidance on electronic health information blocking. This position implies that, in situations involving genetic testing and adolescent health, a physician may reasonably decide on a case-by-case basis—based on professional judgment—to withhold the release of lab results until they have been reviewed (AMA 2021).

#### Practical Steps

A context-sensitive model would require implementing the following policies:

- Establishing a flexible electronic portal system that allows genetic results to be separated from other EHR data. This would involve classifying GI as “sensitive,” in line with other types of adolescent health information—such as that concerning reproductive health, stigmatized mental illness, or substance use—and tagging it accordingly (Norcal Group 2020).

Such segregation is key for maintaining adolescents’ health privacy. The AMA guidance, for instance, acknowledges the challenge of maintaining adolescents’ medical confidentiality by keeping sensitive information hidden/redacted from the EHR when EHRs typically do not segment information based on *who* has access to the patient’s portal, e.g., parent or proxy (AMA 2021).

Segregation is also critical for enabling age-sensitive disclosure of MP. If integration of GI into the EHR is automatic and made fully accessible, physicians and genetic counsellors may not have the opportunity to counsel the minor patient on how to handle such newly acquired, distressing information, or to opt for genetic ignorance. Separating GI from general EHR content allows for more deliberate, developmentally appropriate disclosure.

- a1) Segregation in practice: creating a Genetic Profile within the EHR. An area offering patients genetic testing results should be created, with “educational information written in patient-friendly language for select genetic conditions and pharmacogenetic results,” as was introduced by the PennChart Genomics Initiative (Lau-Min et al. 2021, 604).

b) Differential access to GI, and age-sensitive parental blocking. Adolescents’ differential access to their EHR should be determined by age and testing circumstances and should align with local health and privacy laws, as applied by the HMO responsible for the patient portal and the EHR architecture.

In its 2014 position paper (reaffirmed in 2021), the Society for Adolescent Health and Medicine (SAHM et al. 2014) recommended that EHRs have “flexible functionality to provide differential access to information … for parents and adolescents.” The ideal option suggested was exclusive full access to thirteen-to-seventeen-year-olds, with merely nonconfidential information visible to parents, effectively blocking parents’ access to certain information. Interestingly, SAHM supported parents’ ability “to restrict certain family information from the child’s record” *including GI* such as a family history of Huntington’s disease, and *consanguinity*.

While it is generally recommended that predictive genetic testing of minors (particularly for adult-onset disorders) be deferred until adulthood (ACMG 2013; Botkin et al. 2015; Pereira et al. 2023; Royal College of Physicians. Royal College of Pathologists, and British Society for Genetic Medicine 2022), it could be argued that such information should be made available to adolescents through the EHR in teen pregnancy settings. Such reasoning equally applies to genetic-relatedness information, relevant for adolescents’ reproductive decision-making and potentially carrying future clinical utility (e.g., during prenatal genetic investigations) (ACMG 2013).

As for parental blocking, whereas parents’ access to their pre-adolescents child’s GI should not be blocked, adolescents’ patient portals could be configured to provide limited access to parents, with GI available exclusively to adolescents, subject to counselling (see below) (Norcal Group 2020).

The Canadian Paediatric Society 2023 position statement went further and suggested developing separate patient portals for mature minors (although such transitional technologies are not yet available) (Agostino and Toulany 2023). Such customizable controls for medical information sharing—for parents and adolescents alike—have been found preferrable by healthcare professionals, as they take into account familial circumstances (Hagström et al. 2022b).

c) Mandating pre-disclosure medical counselling for adolescent patients. In the field of medical genetics, where healthcare providers themselves generally lack sufficient genetic literacy, it seems untimely to allow adolescents independent access to GI in their EHR, without physician/caregiver’s support and without proper education and counselling. Minors’ digital nativity, their relative enthusiasm to access patient portals (Hagström et al. 2022b), and the fact that GI may avail itself through DTC genetic testing initiated by them are insufficient to justify exposure to genetic testing results indicating MP without counselling.

In the different, but closely related context of minors requesting confidential health services, the AMA Code of Medical Ethics encourages minor patients to involve their parents (AMA 2012). Nevertheless, it may be exactly in the context before us that adolescents will opt to *withhold* MP news from one or both parents, as such information—implying infidelity or gamete/embryo mix-up—although a burdensome secret to keep, may fracture the family. Furthermore, since adolescents will be *directly* exposed to GI included in their EHR, a fitting opportunity for physicians’/parents’ pre-disclosure involvement may not arise.

We therefore argue that adolescents’ access to health-related and genealogy GI should be conditioned upon receiving medical counselling (without the need for parental consent).

Importantly, counselling should include two separate but complementary specialties:

- psychosocial counselling—to help the adolescent process the finding of MP, ideally, with a case manager assigned to ensure that all aspects of the minor’s welfare are continuously considered; and
- genetic counselling—to explain the clinical significance of sharing non-relatedness information with parents and siblings or withholding it from them.

Our suggested model offers a balanced risk pathway to mitigate the risks of adolescents inadvertently learning about MP through their EHR, while also upholding their autonomy and protecting their health privacy.

## Conclusion

GI is expected to be routinely integrated into the EHR in the near future. However, given the novelty of this issue, policies regarding adolescents’ access to health-related and genealogical GI via patient portals are currently vague and inconsistent.

A comparative review of policies on minors’ access to clinical information in the EHR across different jurisdictions, reveals that some allow minors very early access to their EHR with minimal controls in place, while others implement a stricter policy of deferred or partial disclosure. We were unable to find any current policy explicitly addressing minors’ access to their GI via the EHR/patient portals.

A limitation of our research is the difficulty in evaluating whether inadvertent discovery of genetic genealogical information indicating MP through the EHR, is more, less, or equally harmful to adolescents compared to learning predictive genetic health information. More empirical evidence is needed to inform whether future policy should mirror existing guidance on predictive genetic testing in minors or take a distinct approach.

Further research is also needed to evaluate whether adolescents’ early access to their EHR contributes to their healthcare and psycho-social well-being or enhances their medical literacy in the long-term.

To conclude, inadvertent disclosure of MP is a risk carrying various clinical and ethical implications that should influence the design of access to GI in the EHR. To mitigate this risk and its ramifications, we propose a context-dependent, case-by-case model, that includes informed consent with selectable access options; a flexible EHR system in which GI is classified and tagged as sensitive information and segregated from other health information; differential access to GI with age-sensitive parental blocking; and most importantly, mandatory pre-disclosure medical counselling for adolescent patients.

## Data Availability

No patient data was used in this research.

## Footnotes

1 Govinfo.gov. 45 CFR § 164.524. https://www.govinfo.gov/app/details/CFR-2018-title45-vol1/CFR-2018-title45-vol1-sec164-524/summary.

2 Genetic Information Law, 5761–2000, § 25 & § 26(a) [in Hebrew].

3 Dying Patient Law, 2005 [in Hebrew].

4 See, e.g., ABC v St George’s Healthcare NHS trust [2015] EWHC 1394 (QB) (U.K.); ABC v St George’s Healthcare NHS Trust & Ors [2020] EWHC 455 (QB) (U.K.); Laberge and Burke (2009); Gilbar (2016).

5 It has been argued, on the other hand, that opting for ignorance is inconsistent with being autonomous—being able to control one’s own destiny and to make, through knowledge, rational life choices (Harris and Keywood 2001).
